# Data on the microplastics contamination in water and sediments along the Haraz River estuary, Iran

**DOI:** 10.1016/j.dib.2020.106155

**Published:** 2020-08-07

**Authors:** Nasim Naeeji, Mohammad Rafeei, Hamed Azizi, Marjan Hashemi, Akbar Eslami

**Affiliations:** a School of Public Health and Safety, Shahid Beheshti University of Medical Sciences, Tehran, Iran; b Plastic Department, Processing Faculty, Iran Polymer and Petrochemical Institute, Tehran, Iran; c Environmental and occupational Hazards Control Research Centre, Shahid Beheshti University of Medical Sciences, Tehran, Iran

**Keywords:** Microplastic, Estuary, Haraz river, Sediments, DSC

## Abstract

The type and extent of microplastic contamination in water and sediment of Haraz River were evaluated. Water and sediment samples were collected using a planktonic trawl with a mesh size of 300 µ and a Van Veen grab, from February to May 2017. The morphology and elemental compounds of microplastic particles were analysed using SEM/EDX. Moreover, differential scanning calorimetry (DSC) was done for polymers identification; so that the data could be used for comparative analyses. The number of pieces per kg dry weight in sediments and per cubic meter sampled water was determined. Last but not least, plastic types were also distinguished.

**Specifications table**

**Table:** 

**Subject**	Environmental Science, Pollution
**Specific subject area**	Occurrence and identification of microplastics contamination in water and sediments
**Type of data**	Table Chart Graph Figure
**How data were acquired**	a planktonic trawl and a Van Veen grab sampler, Stereomicroscope, digital scale, SEM/EDX, DSC
**Data format**	Raw and Analysed
**Parameters for data collection**	sampling of water and sediment riverine estuary, Contamination control microscopy, SEM/EDX, DSC analysis
**Description of data collection**	Data of number of particles per cubic meter of water, for a distance of 1.2 km and particles per kilogram of sediment in a 20 cm depth in the foothills of the estuary from February to May 2017. Sampling was carried out in 4 months and each month with two replications. Map of study area. Distribution of MPs by size, color, morphology, shape and types of selected polymers.
**Data source location**	The estuary of the Haraz River located within the Sourkhroud area.
**Data accessibility**	All data is accessible within this article.

Value of the Data

• Microplastic contamination in water and sediments of the Haraz River estuary in 2017 is documented which could be potentially useful and important to the scientific communities and international environmental organizations which providing patterns and maps of marine pollution.
• A benchmark for future studies dedicated to microplastic contamination of an estuarine in Iran is presented which providing Evidence-based data to compare with future studies.
• Haraz River plays an important role in the lives of the people of this region, especially in their agricultural sector.
• The presented information represents the concentration and type of microplastics as emerging pollutants for comparative analysis.Number of particles per cubic meter of water, particles per kilogram of sediment, distribution of microplastics by size, color, morphology, shape and type were monitored and analyzed during 4 months.

## 1 Data

**Fig. 1:**
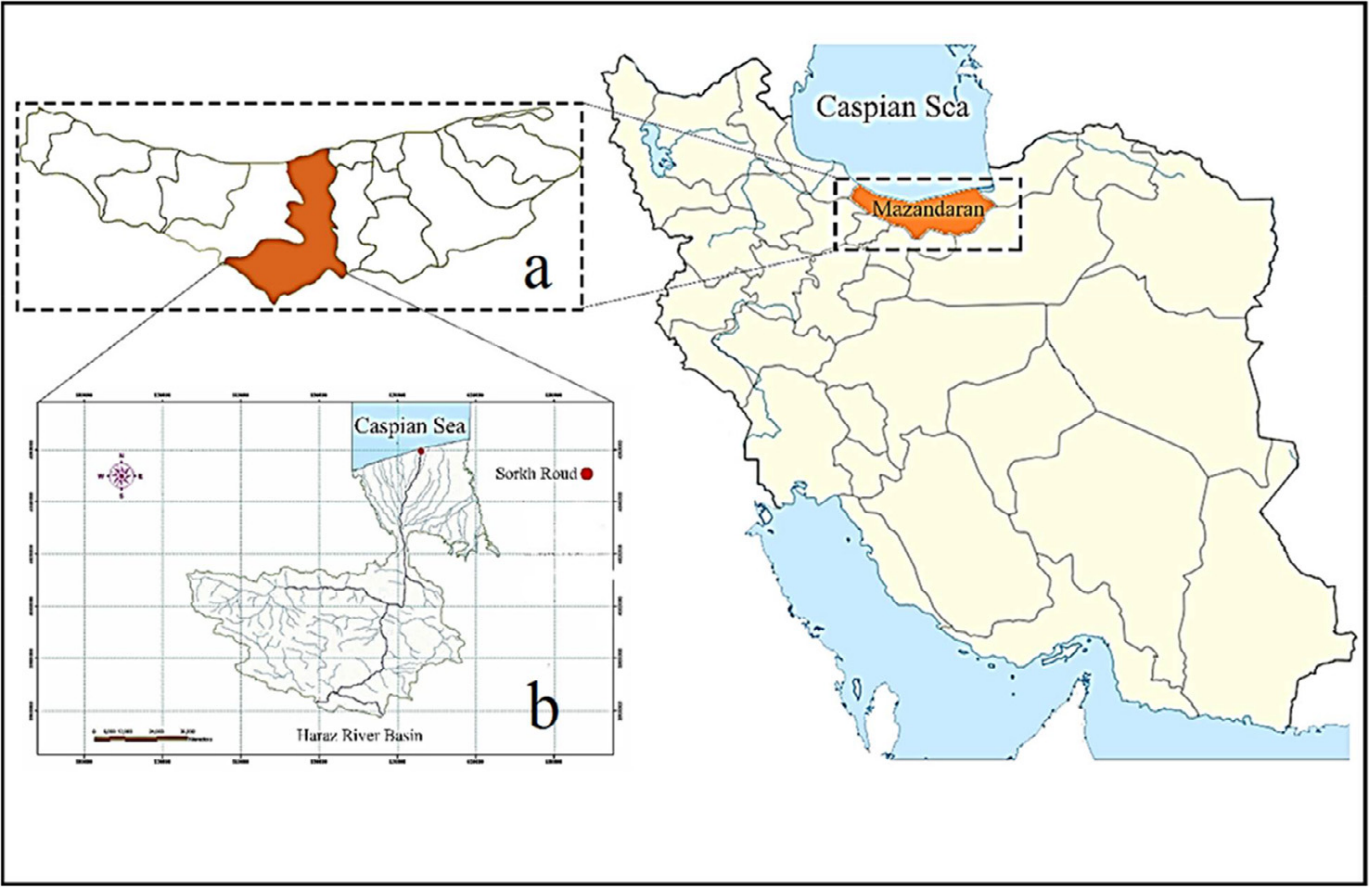
Study area a) Mazandaran province; b) Haraz River basin.

**Fig. 2:**
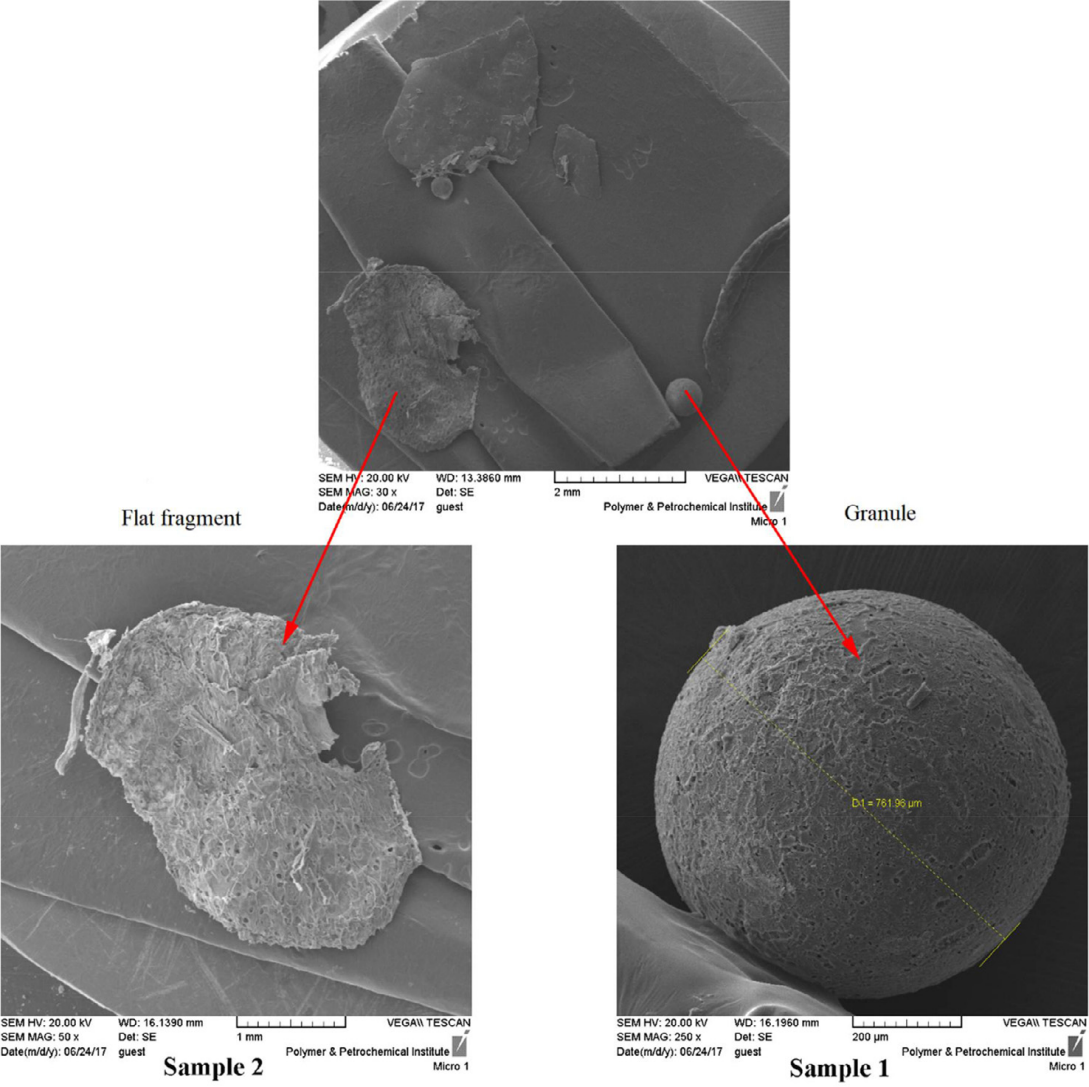
SEM photos of microplastic particles in water sample in February.

**Fig. 3:**
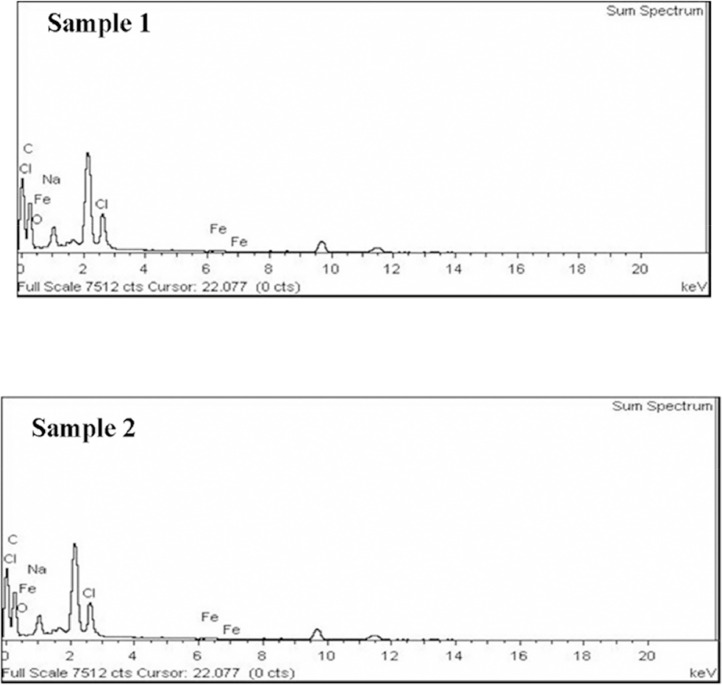
EDX microplastic analysis of Samples 1 and 2 in February.

**Fig. 4:**
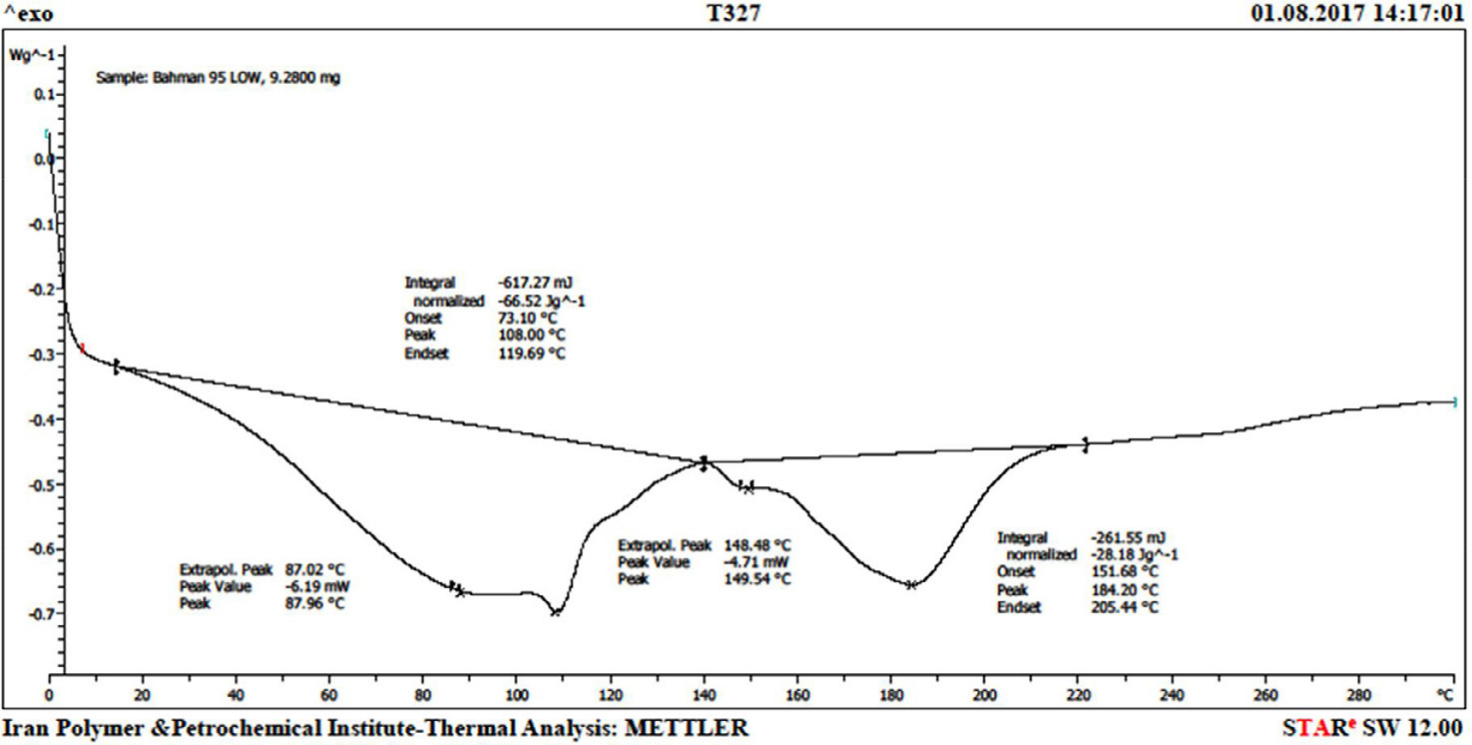
DSC microplastic analysis in February.

The dataset contains information on microplastics contamination in water and sediment samples from the Haraz River estuary during 4 months, from February to April 2017. 299 microplastic particles in 203.52 m^3^ water and 8 microplastic particles from 8 kg sediments were obtained. The morphology and elemental compounds of microplastic particles were analyzed using SEM/EDX. Moreover, differential scanning calorimetry (DSC) was done for polymers identification. Sampling sites (Fig. 1) and SEM photos of microplastic particles of water samples 1 and 2 in February (Fig. 2) are presented. Results of the quantitative and qualitative analysis of microplastics found in water and sediments of Haraz River are provided in (table 1) and (table 2), respectively. Elemental compounds of microplastic found in Samples 1 and 2 in February were analyzed by EDX (Fig. 3); also a DSC microplastic analysis of water sample in February is illustrated in Fig. 4.

**Table 1:** Results of analysis of microplastics found in water of Haraz River.

Month		February	March	April	May
Date		2017/02/03	2017/03/16	2017/04/13	2017/05/13
High density	number	–	–	1.42	–
	Weight (^gr^/_*m*^3^_)	–	–	0.0007	–
	Chemical compounds	–	–	Poly amid12	**–**
	color	–	–	White-blue-green	–
	Type	–	–	–	**–**
Low density	number	1.42	1.97	3.85	3.11
	Weight (^gr^/_*m*^3^_)	0.0014	0.00037	0.0074	0.00158
	Chemical compounds	C, Fe O, Si, K,	C, Fe O, Si, K,	Fe, C O,	C, Fe O, Si, K,
	color	White-blue	White-blue	White-blue-green	White-blue-green-pink
	Type	EVA-LDPE- POM- Polyoxymethylene	EVA-LLDPE-PLA- Polyamid12	EVA-LDPE-PLA- Poly amid12	EVA-LLDPE-PLA

**Table 2:** Results of analysis of microplastics found in sediment of Haraz River.

Month		February	March	April	May
Date		2017/02/03	2017/03/16	2017/04/13	2017/05/13
High density	number	–	–	–	–
	Weight (^gr^/_*m*^3^_)	–	–	–	–
	Chemical compounds	–	–	–	**–**
	Color	–	–	–	–
	Type	–	–	–	**–**
Low Density	number	1	1	4	2
	Weight (^gr^/_*m*^3^_)	0.0021	0.0007	0.0009	0.0008
	Chemical compounds	–	–	c، Fe، o Si، K	–
	color	White	White	White	White
	type	–	–	EVA -LDPE	EVA-LLDPE HDPE

## 2 Experimental design, materials, and methods

Water and sediment samples from the Haraz River estuary were collected using trawl and grab. Sampling was carried out in 4 months and each month with two replications. The volume of water passing through the trawl was 25.44 m^3^ in each sample time. Microplastic particles were visually separated from the collected waste of 8 samples.

### 2.1 Collection of samples

Water and sediment samples from the Haraz River estuary were collected using trawl and grab. Sampling was carried out in 4 months and each month with two replications. For water sampling, a 52 cm wide planktonic trawl with a mesh size of 300 µ was used [1]. The trawl was driven by a boat from the river estuary in the opposite direction of water flow, for a distance of 1.2 km. The materials were collected according to the size of the trawl pores. The retained debris within the trawl was washed using site water and placed inside a labeled container. It was then prepared for processing and analysis. Sediment sampling was carried out using a 20 cm × 20 cm Van Veen grab [2, 3]. The sediments were collected from a 20 cm depth in the foothills of the estuary.

### 2.2 Methods

#### 2.2.1 Water sample preparation

The water samples were first passed through a 5 mm stainless steel sieve, and then again through a 300 µ sieve. After that, they were washed off using deionized water, transferred into 1000 mL pre-weighed glass beakers, and placed into an oven at 75 °C, for approximately 24 h. After recording the dry weight, 20 mL of a solution of 0.05 M Fe (II) (FeSO_4_•7H_2_O) and 20 mL of 30% hydrogen peroxide solution (H_2_O_2_) were added to facilitate chemical digestion of the labile organic material. After washing with deionized water to remove residual H_2_O_2_, the contents of the sieve were poured into a decanter [3, 4]. Density separation was carried out by means of NaCl hyper saline solution (300 g/L) [3], [4], [5], [6]. Afterwards, the presence or absence of the microplastic particles was investigated through visual inspection [4,7] and by using a stereomicroscope [8].

#### 2.2.3 Sediment sample preparation

The sediment samples were passed through a 5 mm sieve and then a 300 µ sieve. Subsequently, they were rinsed with deionized water, dried in the oven at 90 °C for 24 h. Then, 1 kilogram of dry sediment was transferred to a 2000 mL beaker containing saturated NaCl solution (with a density of 1.2 kg/L) [3, 8, 12]. Particles with lower density floated on the surface were collected using a 300 µ sieves and transferred into a petri dish; then dried at the ambient temperature [3].

### 2.3 Analytical techniques

The number of microplastic particles collected in each trawl was determined by counting the total number of collected particles. The final concentration of microplastic particles was determined by mass per unit volume (g/m^3^) and particle per unit volume (particles/m^3^) [4].

In order to diagnose the morphology of the microplastic particles, The Scanning Electron Microscope (SEM) examination was carried out to characterize its surface composition [5, [13], [14], [15]]. Twelve microplastic particles with different shapes were randomly selected from the samples and analyzed using SEM. The microplastic particles found were of different shapes, such as regular or irregular spherical particles, flat fragments, films, and fibers (Fig. 2).

The quality of the elements forming the microstructures (chemical characteristics) was determined using the Energy-Dispersive Spectroscopy (EDX) analysis [8]. EDX analysis showed the presence of carbon, chlorine, iron, sodium, aluminum, potassium, calcium, silica, and oxygen in the microplastic particles of samples (table 1) and (table 2).

Differential scanning calorimetry (DSC) analysis was used for qualitative analysis of different types of microplastic polymers according to their melting properties [9], [10], [11]. According to the DSC analysis, the diversity of polyethylene covinyl acetate (EVA) and low density polyethylene (LDPE) polymers were higher than that of the other polymers.

## Declaration of Competing Interest

The authors declare that they have no known competing financial interests or personal relationships that could have appeared to influence the work reported in this paper.

## Appendix Supplementary materials

Image, application 1

Image, application 2
