# Supplementary material for: Data on the microplastics contamination in water and sediments along the Haraz River estuary, Iran
Source: Data Brief. 2020 Aug 7;32:106155. doi: 10.1016/j.dib.2020.106155 (PMC7452695; doi:10.1016/j.dib.2020.106155)
Supplement: Supplementary file 2 [file mmc2.docx]

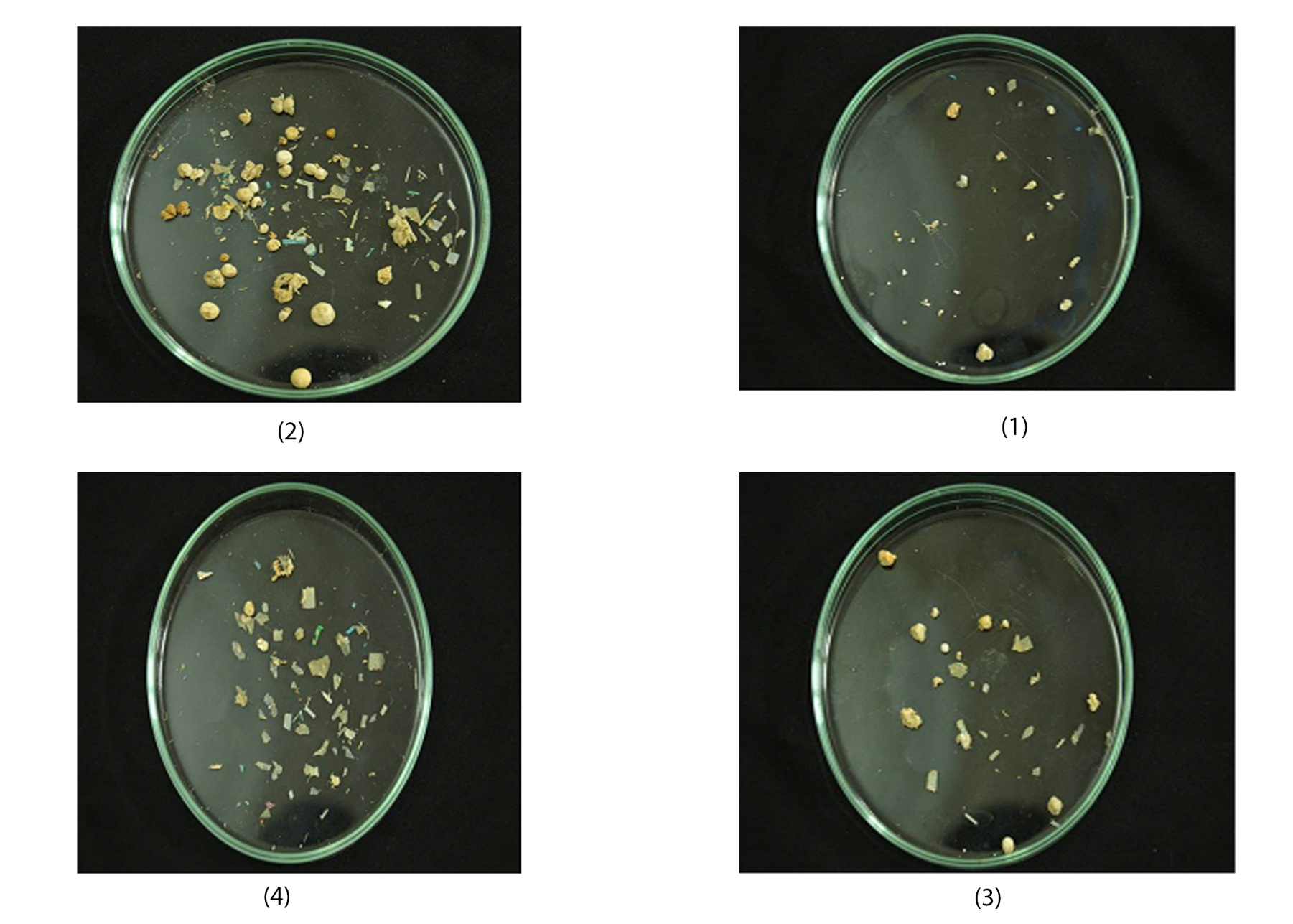


Fig. 3. Pictures of microplastic particles of water samples

(1) Low density microplastic sampled in March (2) Low density microplastic sampled in February;

(4)Low density microplastic sampled in May; (3) High density microplastic sampled in February


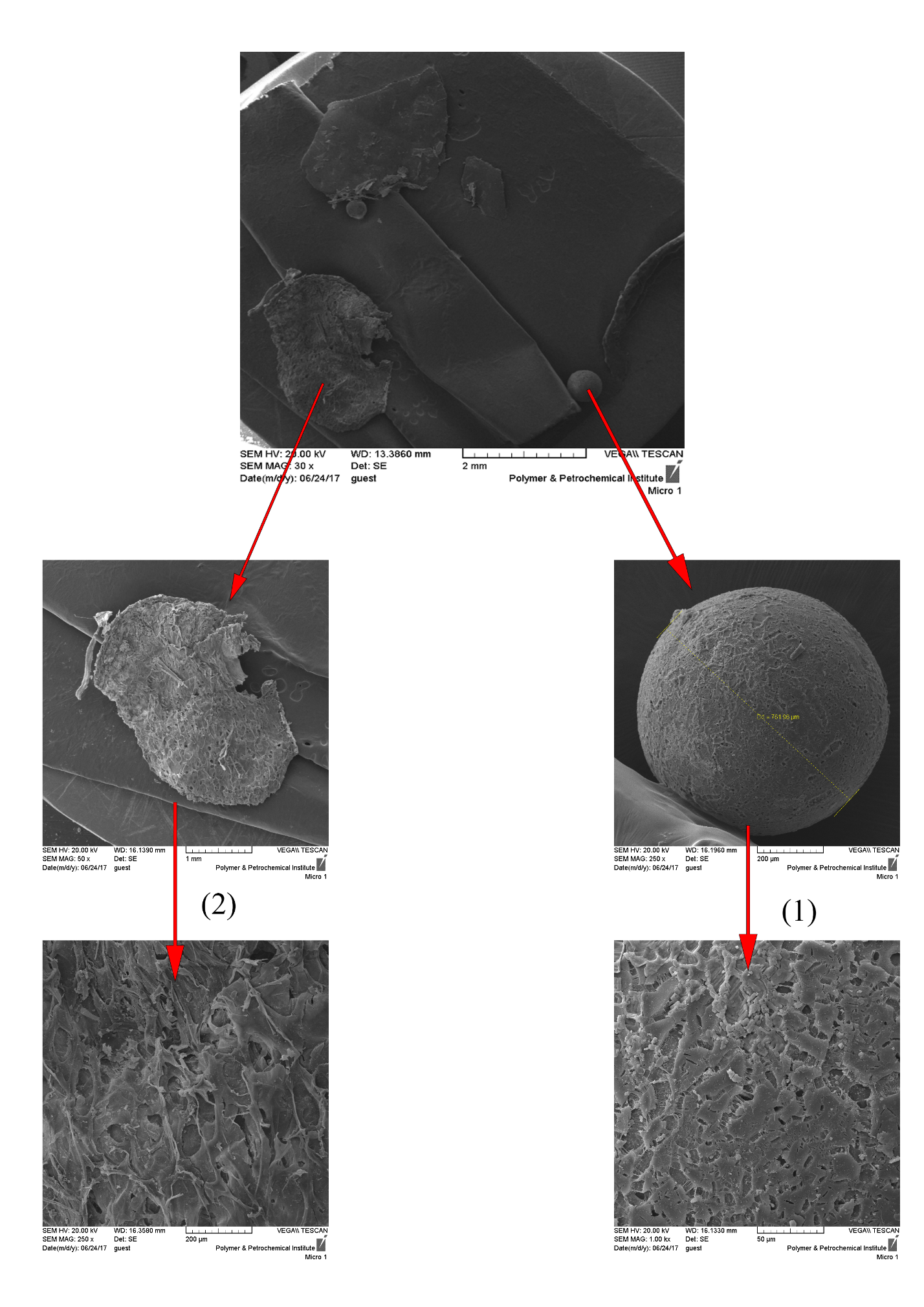


SEM photos of microplastic particles in water sample in February

SEM photos of microplastic particles in water sample in February


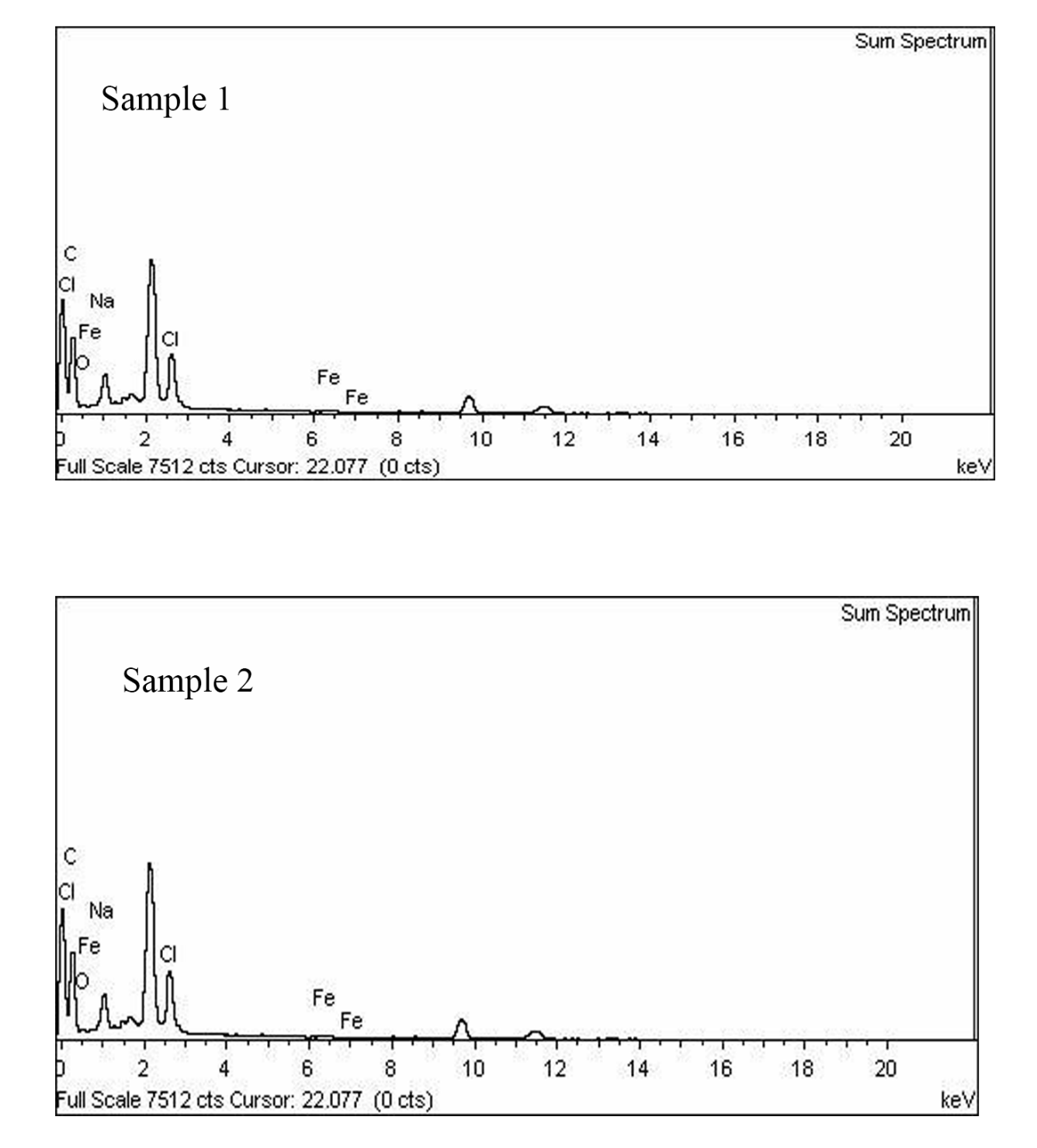


EDX analysis of Sample 1 and 2 microplastic particles in water in February


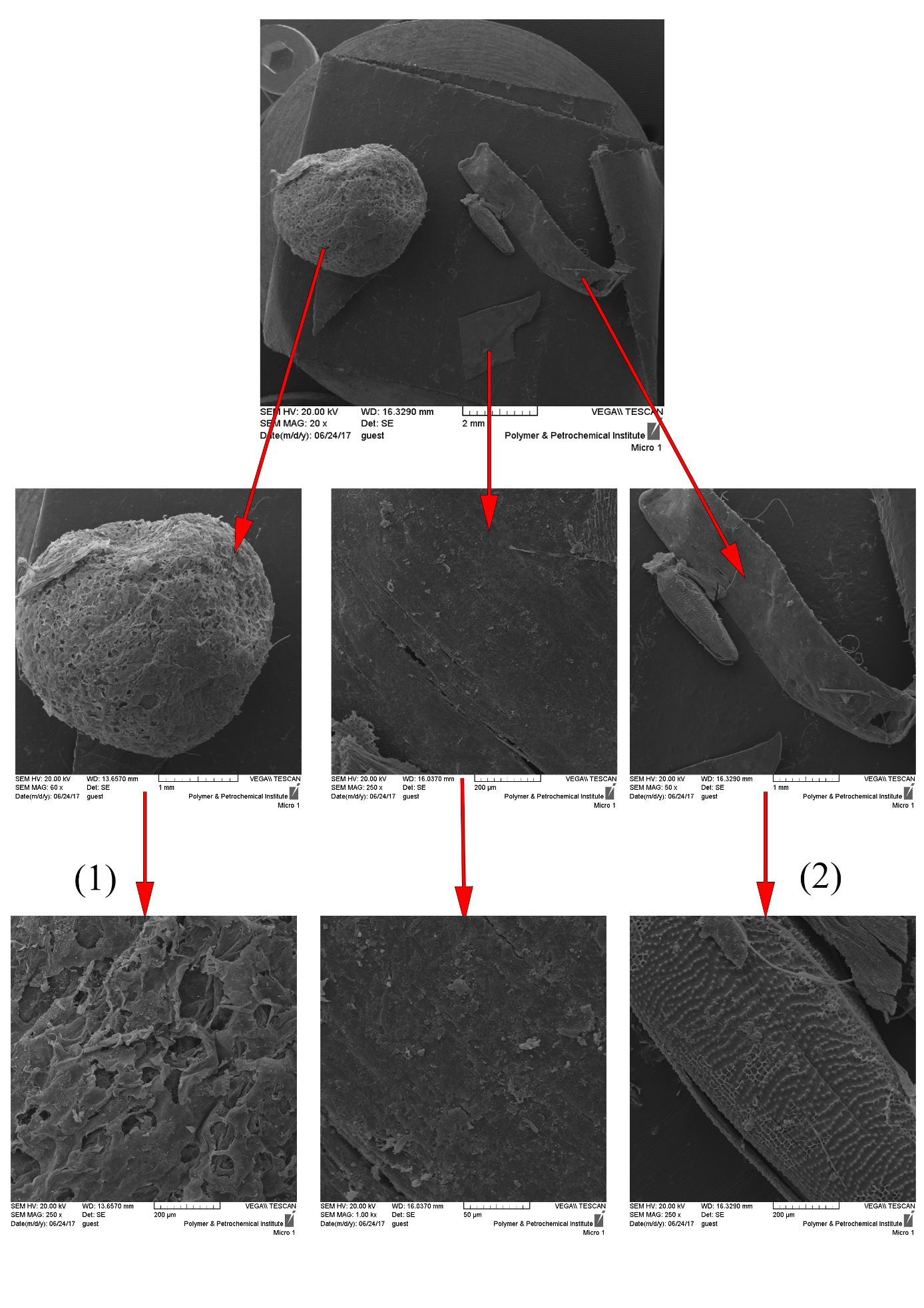
SEM photos of microplastic particles in water sample in March


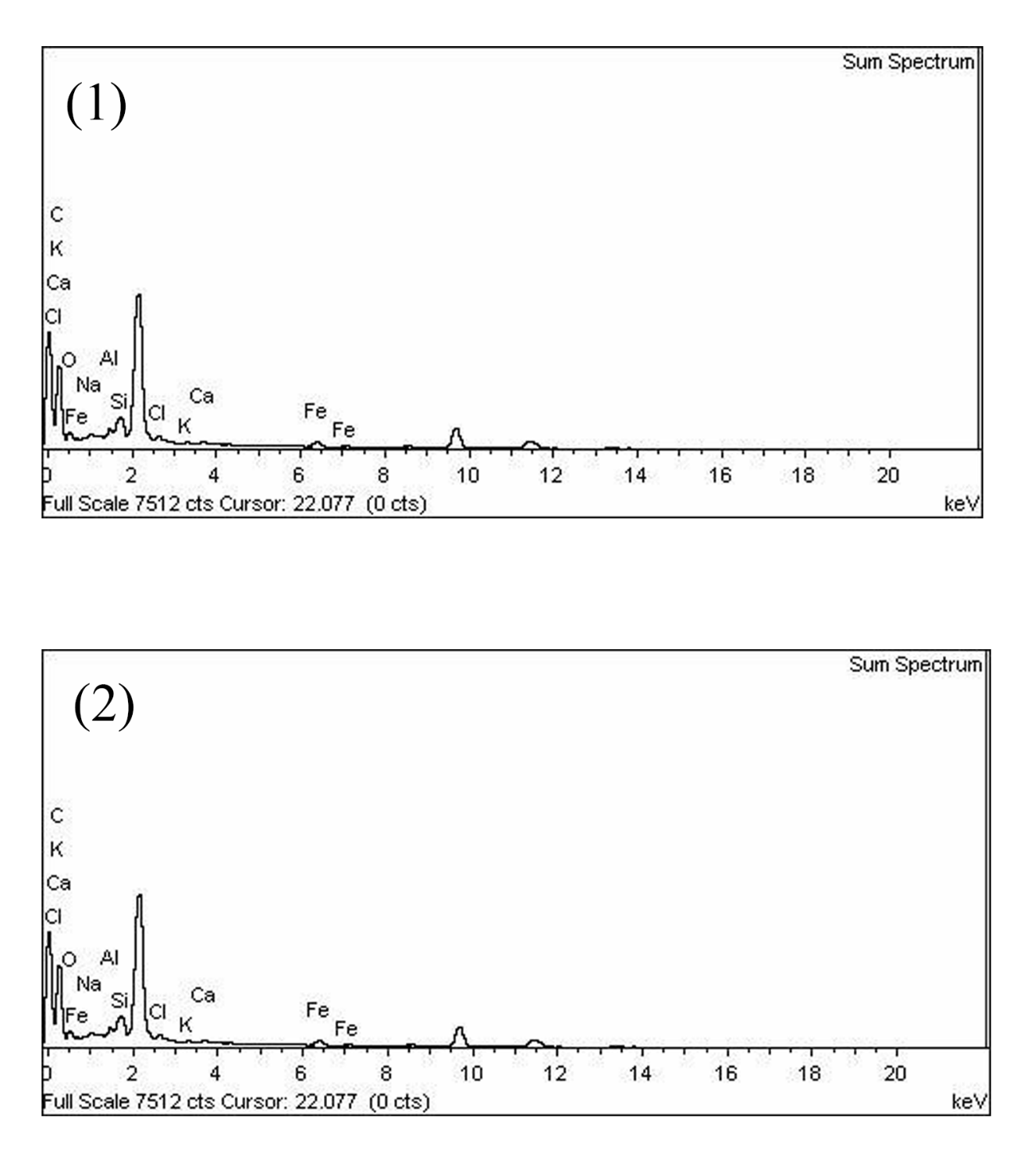


EDX analysis of Sample 1 and 2 microplastic particles in water in March


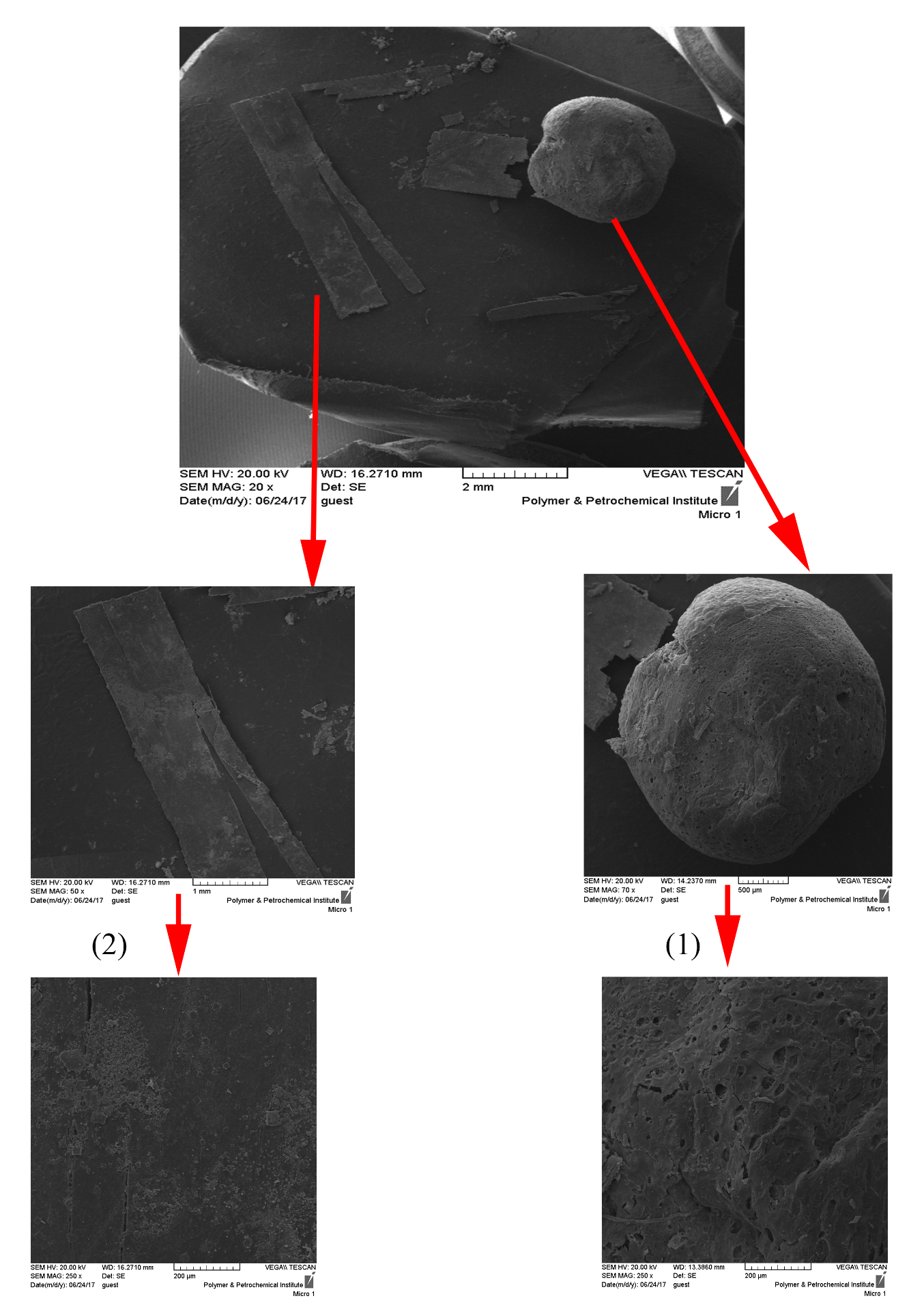


SEM photos of microplastic particles in water sample in April


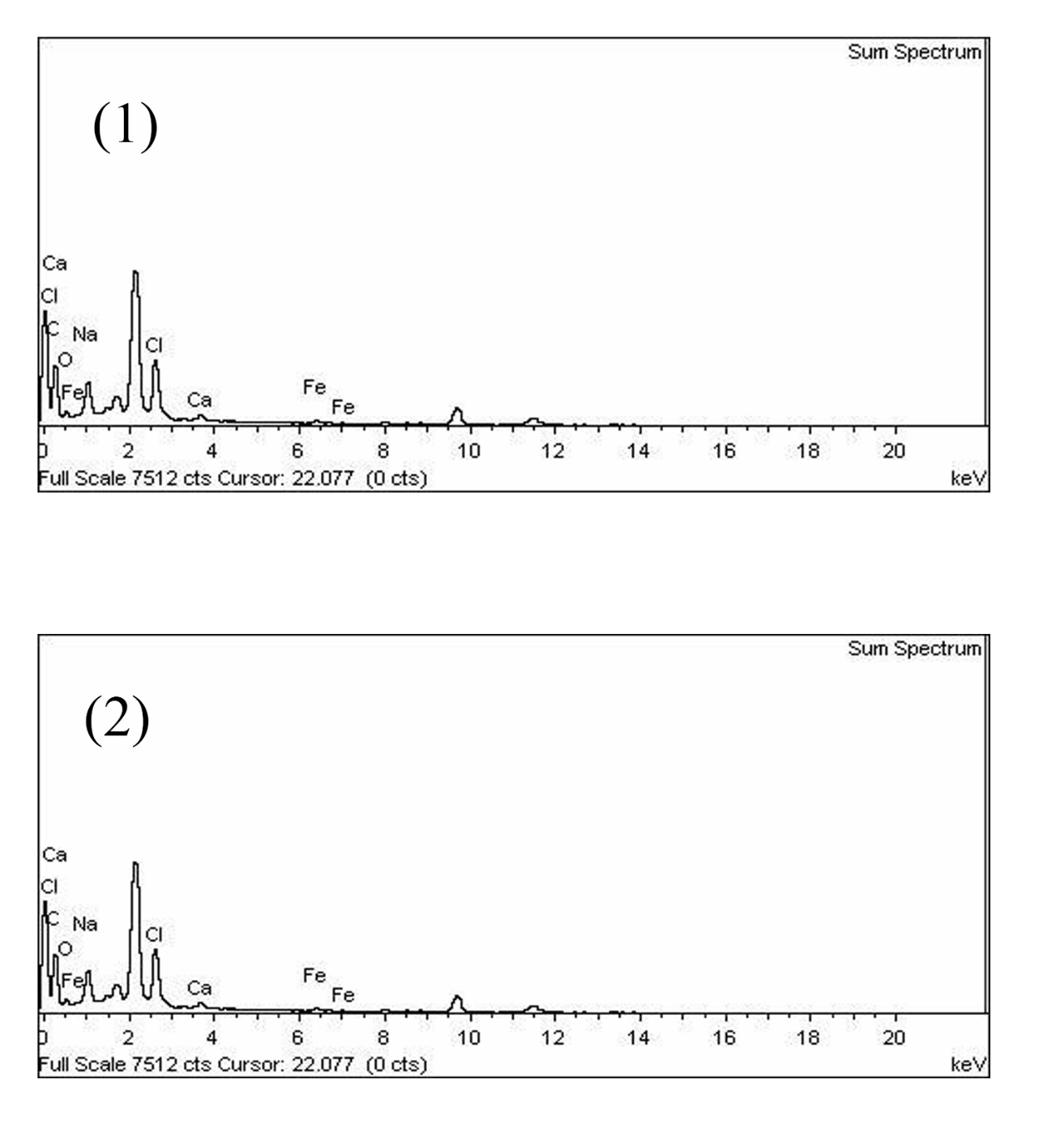


EDX analysis of Sample 1 and 2 microplastic particles in water in April


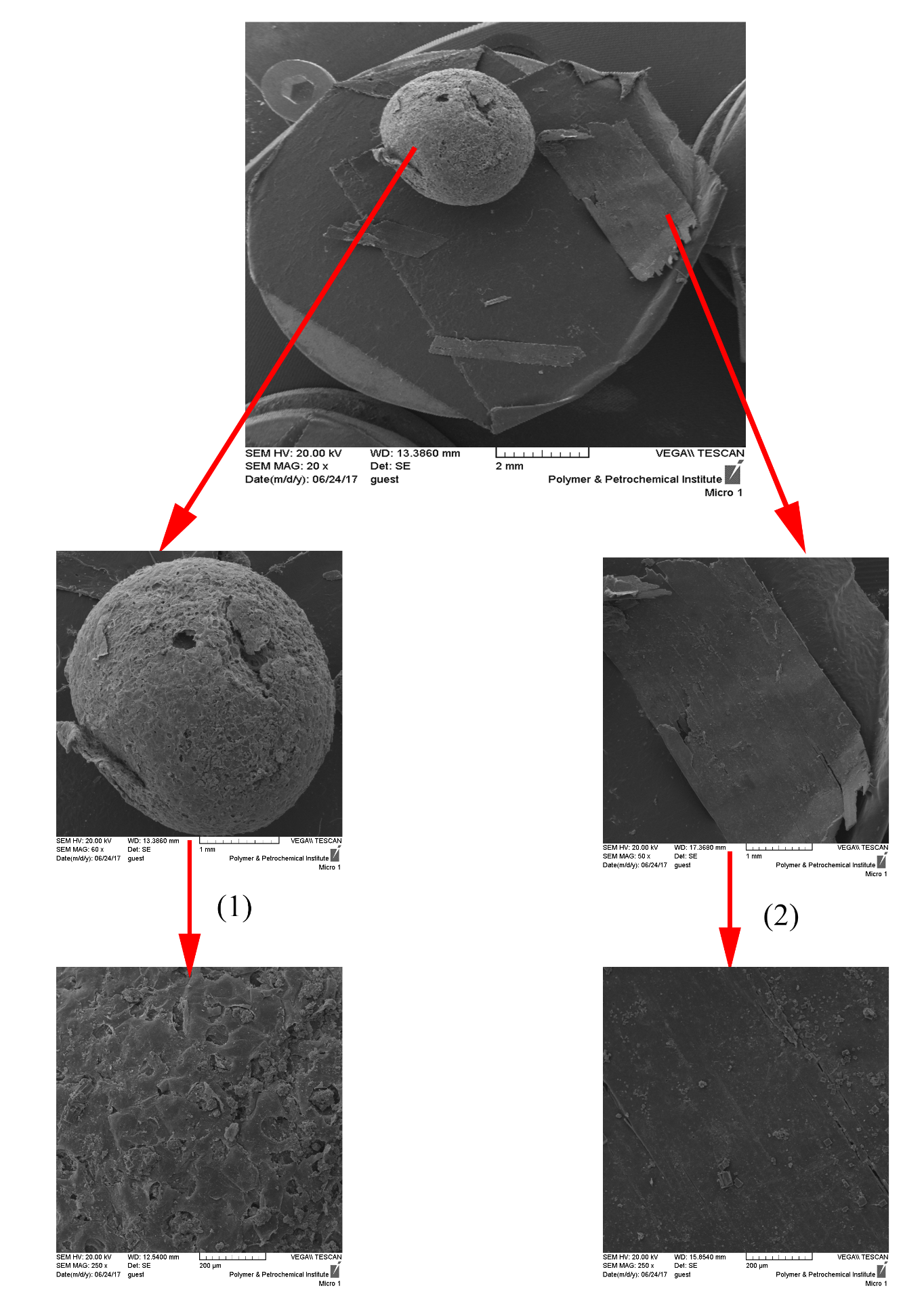


SEM photos of microplastic particles in water sample in May


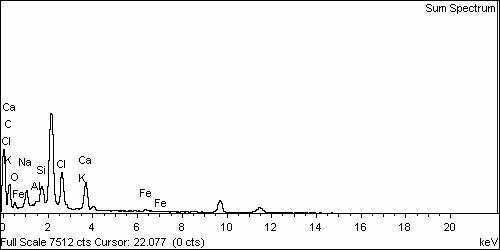


EDX analysis of Sample 1 microplastic particle in water in May


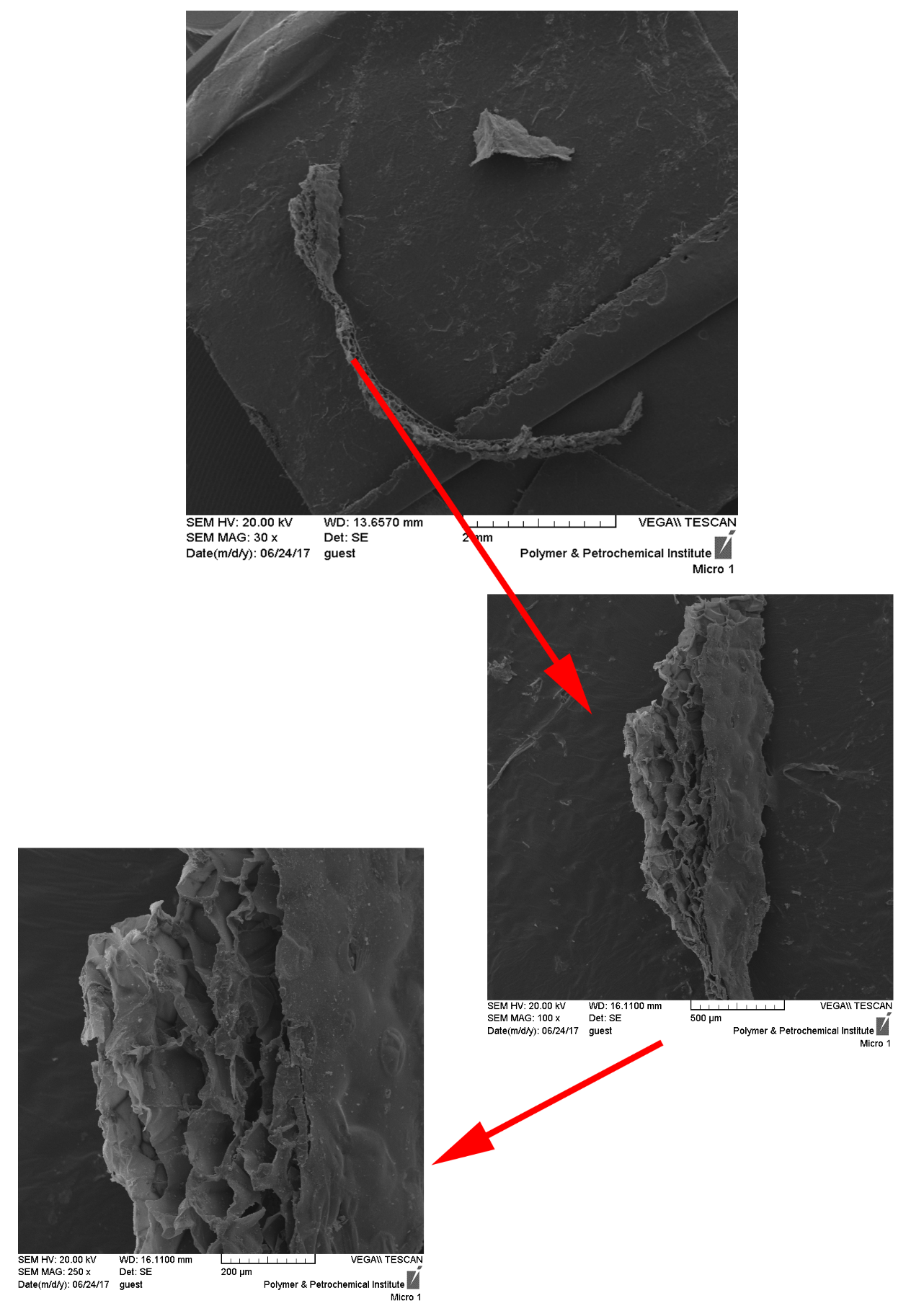


SEM photos of microplastic particle in sediment sample in May


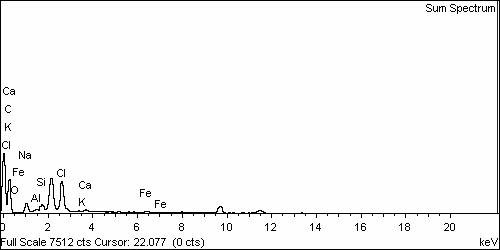


EDX analysis of microplastic particle in sediment sample in May


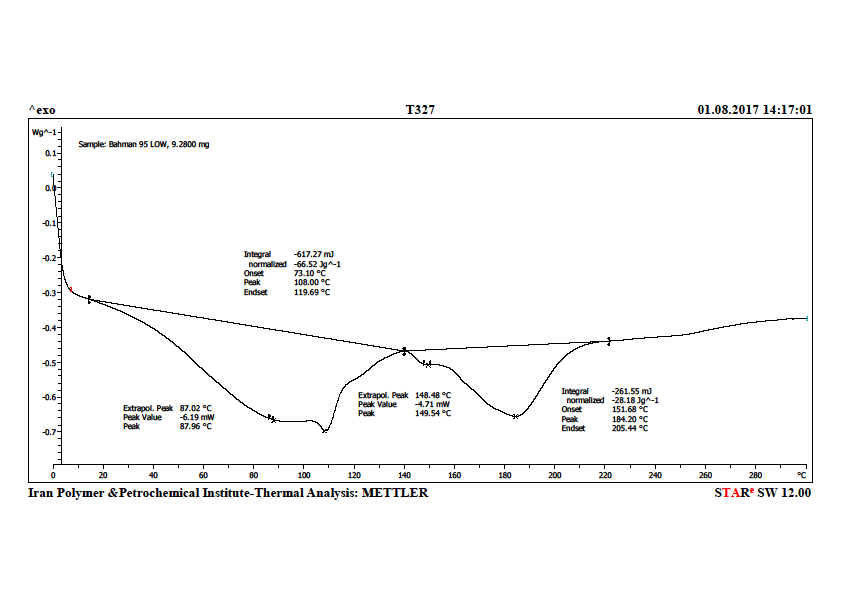


DSC microplastic analysis of water sample in February


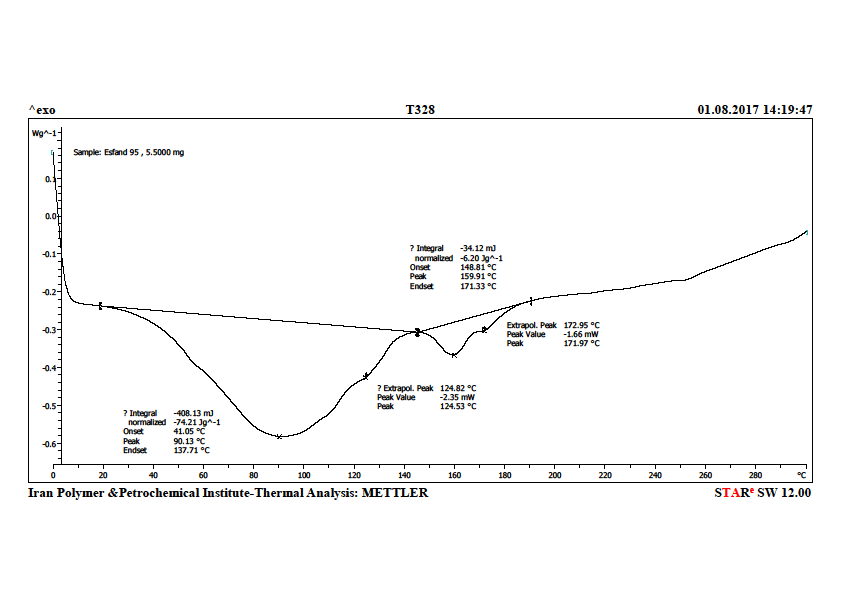


DSC microplastic analysis of water sample in March


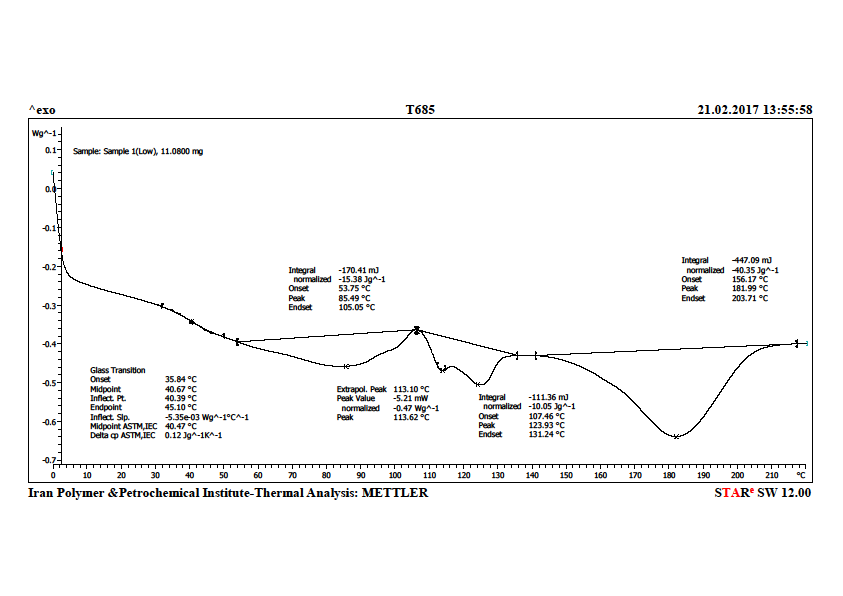


DSC analysis of low density microplastic of water sample in April


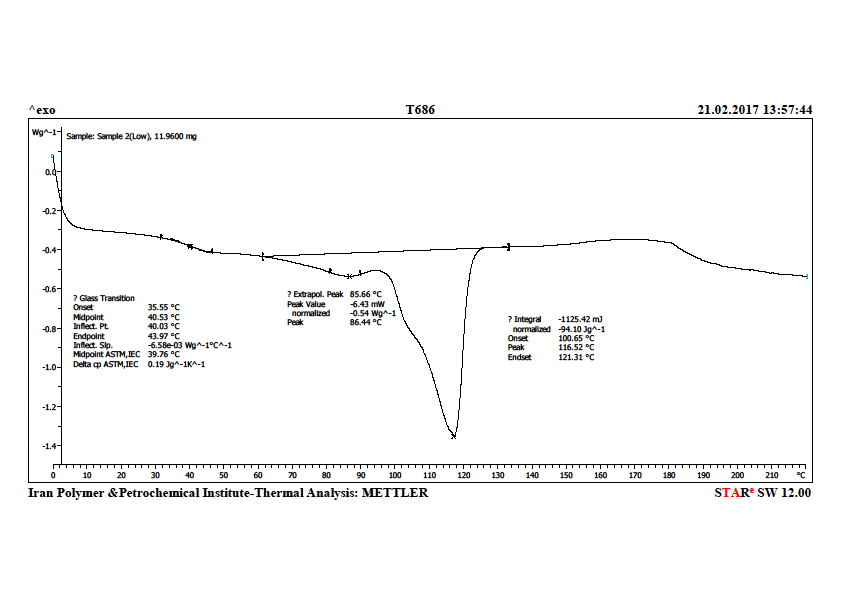


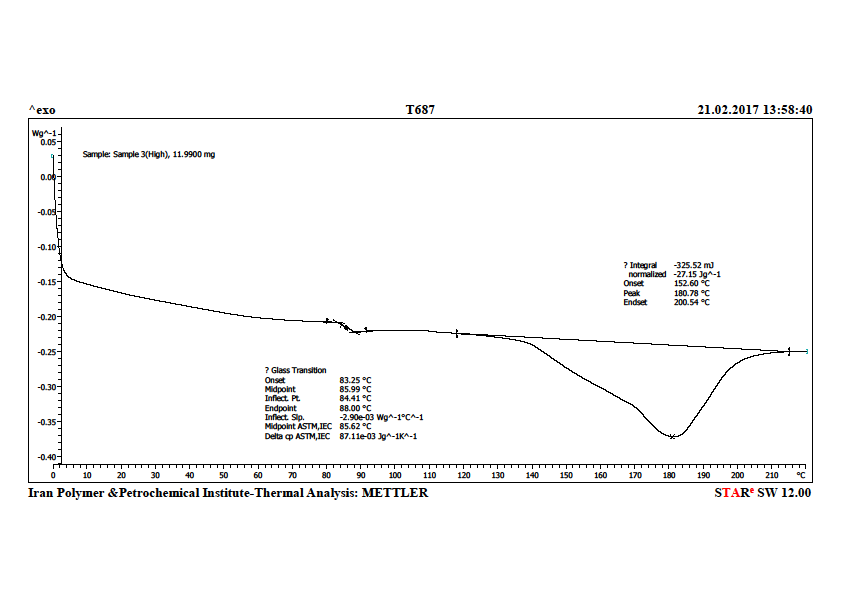
DSC analysis of low density microplastic of water sample in April

DSC analysis of high density microplastic of water sample in April


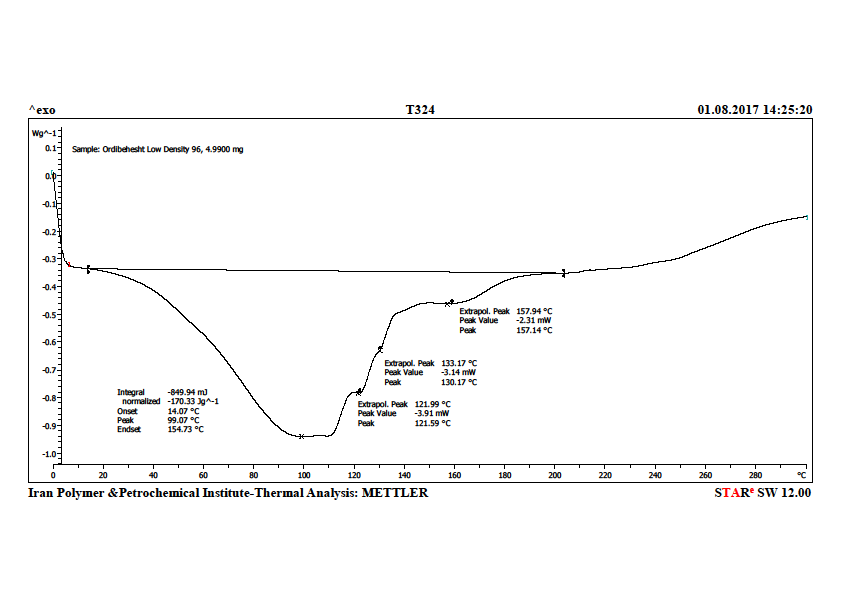


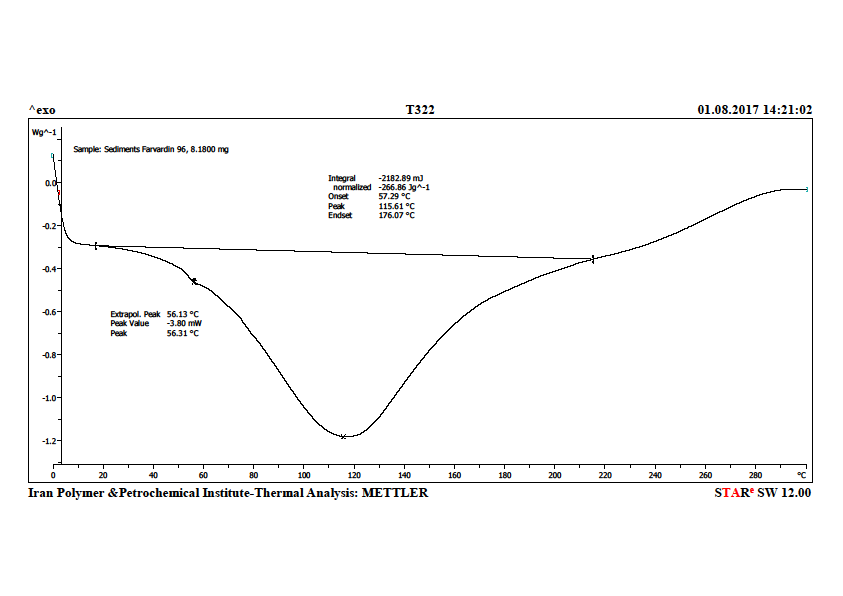
DSC analysis of low density microplastic of water sample in May

DSC analysis of microplastic of sediment samples in April


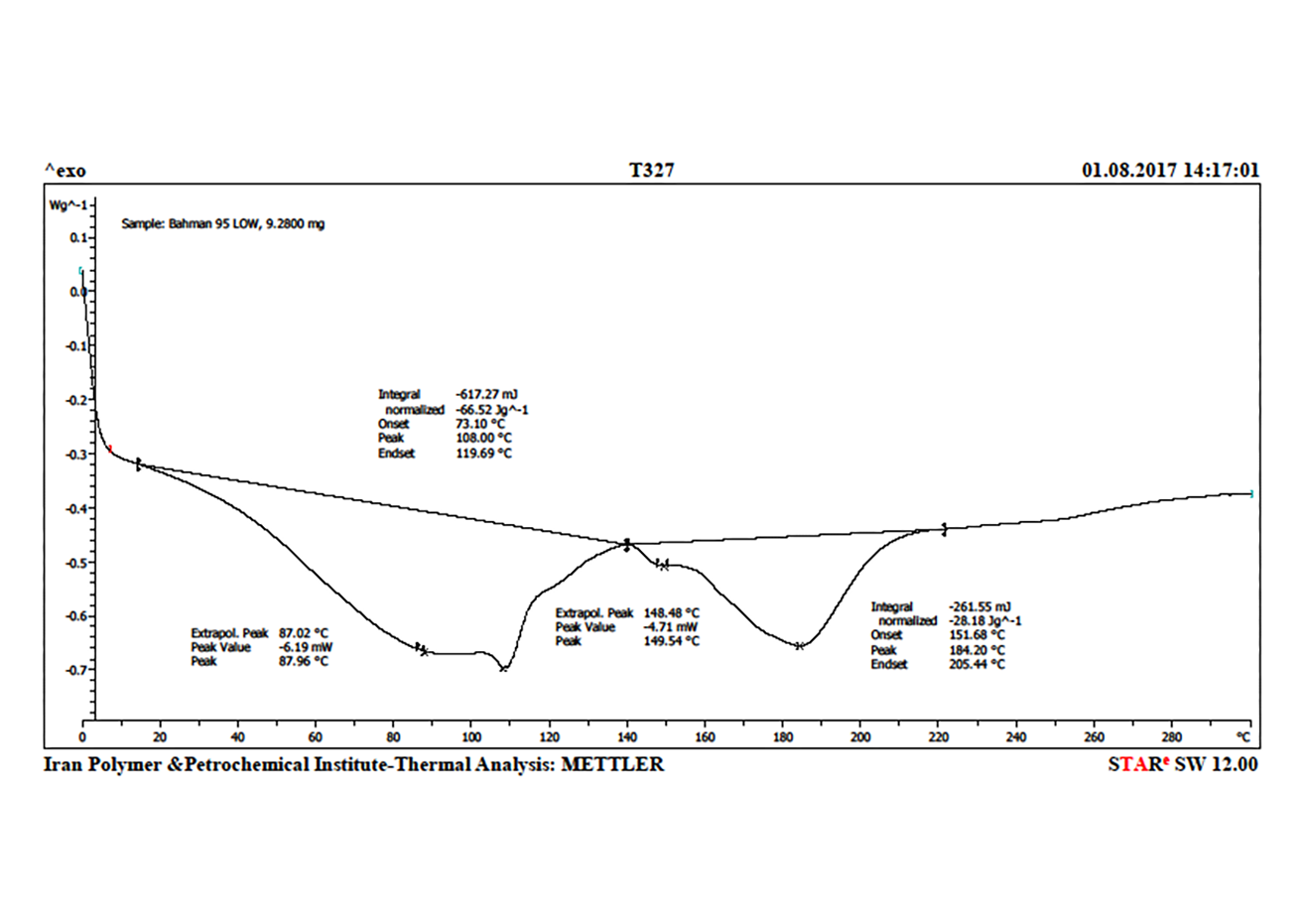


DSC analysis of microplastic of sediment samples in May
